# Exosomes in Osteoarthritis: Breakthrough Innovations and Advanced Tissue Engineering for Cartilage Regeneration Since 2020

**DOI:** 10.3390/biomedicines13102486

**Published:** 2025-10-13

**Authors:** Xiao-He Yang, Shu-Yin Chen, Quan-Fa Zhou, You-Zhi Cai

**Affiliations:** 1 Department of Orthopedic Surgery, the First Affiliated Hospital, Zhejiang University School of Medicine, 79 Qingchun Road, Hangzhou 310008, China; 2 Institute of Sports Medicine, Zhejiang University, Hangzhou 310008, China

**Keywords:** exosome modification, OA therapy, cartilage regeneration, stem cells, tissue engineering

## Abstract

Background/Objectives: Osteoarthritis (OA) is a prevalent age-related degenerative joint disease causing cartilage damage, leading to a debilitating lifestyle. However, there are currently no drugs on the market that promote cartilage repair, and advanced cases often require arthroplasty. Increasing evidence suggests that exosomes, the smallest extracellular vesicles (30–150 nm) secreted by all cell types, are involved in the pathological process of OA and play a crucial and complex role in its progression. This review aims to provide in-depth insights into exosome biology, isolation techniques, their role in OA pathophysiology, and their clinical therapeutic potential. Methods: We systematically reviewed studies published since 2020 on exosomes in OA, focusing on their biological properties, isolation techniques, pathological roles, and therapeutic applications. Results: Exosomes derived from synovial fluid, chondrocytes, synoviocytes, and mesenchymal stem cells regulate key processes in OA progression, including inflammation, apoptosis, extracellular matrix degradation, and regeneration. Various cell-derived exosomes show therapeutic potential for cartilage damage/OA. However, their mechanisms of action have not been fully investigated. Moreover, emerging methodologies, such as utilizing novel materials for exosome delivery, potentially facilitate the development of more effective and personalized therapeutic interventions. Conclusions: Exosomes exert dual roles in OA pathogenesis and therapy. Although challenges remain regarding their sources, dosage, delivery, and standardization, exosome-based strategies represent a promising cell-free therapeutic approach with potential applications in personalized and precision medicine.

## 1. Introduction

Osteoarthritis (OA) is a progressive disease characterized by articular cartilage degeneration, synovial inflammation, and subchondral bone remodeling, which causes a huge social and economic burden [1]. Chronic inflammation promotes cartilage degeneration during OA progression, but the specific molecular mechanisms remain unclear. A large amount of evidence shows that exosomes, as extracellular vesicles (EVs), play a role in tissue–tissue and cell–cell communication in homeostasis [2] and disease [3], and the mechanism of exosomes involved in the occurrence and progression of OA has been reported in recent years. The development of OA is recognized to be associated with a degradation–synthesis imbalance of chondrocytes, extracellular matrix and subchondral bone. Thus, understanding how exosomes contribute to these processes will help to develop novel therapeutic strategies of OA.

Treating OA has proven challenging since articular cartilage lacks a blood supply while the chondrocytes are highly differentiated cells with limited capacity for migration and proliferation [4]. Common medical treatments include acetaminophen and other nonsteroidal anti-inflammatory drugs (NSAIDs), opioids, local analgesics, corticosteroid injections, and hyaluronic acid injection. These therapies, however, are unable to alter the degenerative process or restore articular cartilage regeneration [5]. For individuals with severe OA, surgical joint replacement can enhance function and quality of life in the long-term. The most frequent restrictions are joint instability and infection, which necessitate additional joint revision surgery, particularly in individuals who are overweight [6]. In recent years, stem cell treatment, especially mesenchymal stem cell (MSC) treatment, has advanced quickly in the field of OA research and regenerative medicine. However, clinical applications of stem cells have attracted considerable attention, such as teratomas, immune rejection, and dose-dependent effects requiring several cells [7,8]. Numerous investigations have demonstrated that MSCs primarily use paracrine signals to alter cells, particularly through the exosomes they generate [9,10]. Therefore, exosome-based therapy offers a potential “cell-free” treatment for OA and may be a successful substitute for stem cell therapy. In this article, we review the biological characteristics of exosomes, particularly in the pathological process of OA, and the research progress of exosomes as an advanced treatment for OA.

## 2. The Biological Characteristics of Exosomes in the Deterioration of OA

**Figure 1:**
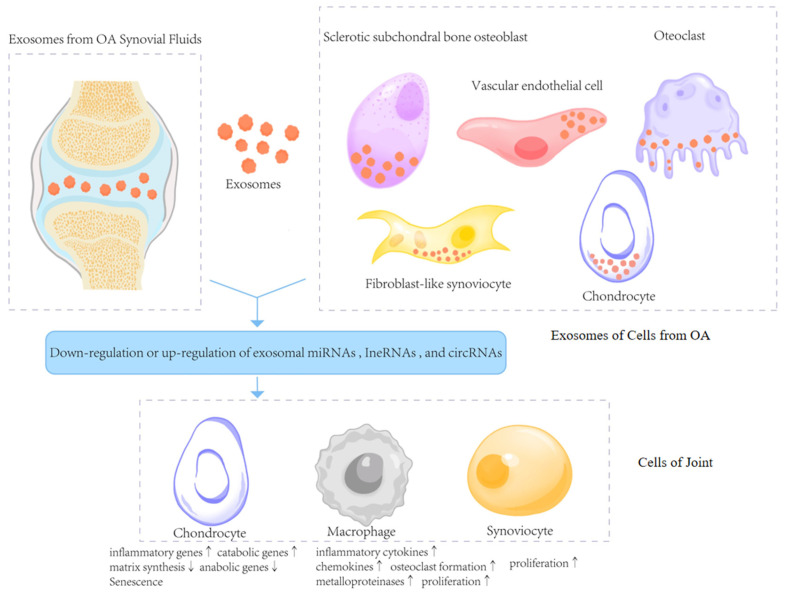
Exosomes in the deterioration of OA. This figure illustrates how exosomes derived from the synovial fluid and joint cells in OA carry ncRNAs such as miRNAs, lncRNAs, and circRNAs that are either upregulated or downregulated. These exosomes are taken up by recipient cells including chondrocytes, macrophages, and synoviocytes, resulting in altered gene expression, inflammatory signaling, extracellular matrix degradation, cellular proliferation, and senescence (↑ indicates upregulation/increase; ↓ indicates downregulation/decrease). These changes promote cartilage degeneration and joint inflammation in OA progression.

As a subtype of EVs, exosomes are membrane-bound vesicles, with a diameter of 30 to 150 nm, released by cells in all living systems under both physiological and pathophysiological conditions [11]. The joint is mainly composed of cartilage, subchondral bone and joint capsule. A variety of pathologic factors participate in the destruction of the homeostasis of the intra-articular microenvironment. The synovial tissue of OA patients showed a higher number of immune cells which expressed pro-inflammatory cytokine, such as tumor necrosis factor (TNF)-α, interleukin (IL)-1β, IL-6, and IL-22 [12]. It has been suggested that matrix metalloproteinase-13(MMP-13) is a key enzyme responsible for degenerative changes in cartilage, which aids in the degenerative process that takes place throughout the pathophysiology of OA [13]. Therefore, investigating the connection between exosomes and these pathogenic elements should improve our comprehension of OA pathology and identification of novel biomarkers and therapeutic targets for OA treatment (Figure 1).

Comprehensive profiling of exosomes derived from joint tissues and fluids has demonstrated that they are enriched in ncRNAs, including microRNAs (miRNAs), long non-coding RNAs (lncRNAs), and circular RNAs (circRNAs) [14]. Exosomal ncRNAs from different joint cells, such as chondrocytes, MSCs, and fibroblast-like synoviocytes (FLSs), alter the proliferation, apoptosis, inflammatory response and differentiation, and abilities of target cells, therefore potentially disrupting joint tissue homeostasis and affecting the development of OA [15,16,17,18]. In this section, we summarize the recent studies on the specific roles and mechanisms of exosomal miRNAs, lncRNAs, and circRNAs in the progression of OA (Table 1).

### 2.1. Exosomes in Synovial Fluids

Synovial fluid (SF) is directly and intimately related to the synovial membrane, articular cartilage and other joint tissue types, which makes it useful for tracking pathological alterations in the joint area. Numerous studies have shown that exosomes from synovial fluid play a role in the pathological process of OA. Ji et al. discovered that OA patients had a significantly lower expression of miR-182-5p and its target, tumor necrosis factor α-induced protein 8 (TNFAIP8) from the synovial fluid exosomes. The reduction in exosomal miR-182-5p led to a suppression of chondrocyte proliferation, migration and invasion, while it downregulated the miR-182-5p rescuing the OA degeneration [42]. Furthermore, Semerci et al. investigated another essential exosomal miRNA, miR-127-5p, in cartilage differentiation. They validated the role of miR-127-5p in cartilage regulation and compared the expression profiles between human adipose tissue-derived mesenchymal stem cells (hAT-MSCs) and human synovial fluid-derived mesenchymal stem cells (hSF-MSCs) during chondrogenesis in relation to cartilage regenerative treatment. They discovered that hAT-MSCs are superior sources of miR-127-5p than hSF-MSCs for promoting chondrogenesis, thus may contribute to the identification of mechanisms responsible for chondrocyte development and the advances of therapeutic methods [43]. Huang et al. widely compared the proteomics of SF-derived exosomes in OA with several other inflammatory arthritis (IA) patients. They found twenty-eight protein profiles, such as cartilage intermediate layer protein 1 (CILP1) and stromal interaction molecule 1 (STIM1) were highly expressed in OA patients [44]. Lai et al. took a further step and investigated the effect of synovial fluid-derived exosomal miRs on inflammation, survival, proliferation of chondrocyte in correlation with cartilage degeneration. They discovered that miR-214-3p exosomes could reduce chondrocyte inflammation and degeneration of cartilage tissues [45]. Yang et al. discovered that the exosomes, which protect LncRNAs from RNase degradation, could provide accessible options for biomarkers. Among the biomarkers, NONRATT018001.2 and MSTRG.73954.4 showed the most reliable potential for distinguishing different types of organ injury induced by drugs, particularly enabling early prediction of tissue injury [46].

The majority of cytokines in synovial fluid, according to Gao and colleagues, are not only present in free form but are also concentrated in exosomes. Compared to the cytokine profiles of the soluble synovial fluid, exosomes from individuals with end-stage OA have increased levels of cytokines, particularly chemokines [47]. Furthermore, Wu et al. compared the cytokine profiles such as lncRNAs, circRNAs, and mRNAs, from synovial exosomes between OA and control patients [48]. Osteoarthritis exosomes and control group synovial exosomes showed differential expression of 52 mRNAs, 196 lncRNAs, and 98 circRNAs. There are 22 miRNAs, 45 lncRNAs, and 34 circRNAs enriched in the PI3K/Akt and autophagy pathways correlated with 7 mRNAs, according to an intersectional analysis of the ceRNA network [48]. These potential biomarkers and hypothetical molecular in synovial fluids may provide helpful diagnostic and therapeutic insights for OA treatment.

### 2.2. Exosomes in the Tissues of OA

Exosomes are not only reported in the SF, but also in the synovial membrane, articular cartilage and other joint tissues of OA. According to Wu et al., synovium-specific exosome containing miR-182, which was significantly increased in the synovial tissue of advanced OA, can modulate inflammation and apoptotic signaling [49]. Synovium-derived miR-182 not only regulates inflammatory response in the early stage of OA by regulating the expression level of forkhead box O-3 (FOXO3) but is also involved in the apoptotic signal in severe OA. As for the end stage of OA, Wu et al. discovered that chondrocytes internalized exosomes produced from osteoblasts in the sclerotic subchondral bone, which in turn triggered catabolic gene expression and reduced the expression of chondrocyte-specific marker [50]. MiR-210-5p, which is substantially concentrated in OA sclerotic subchondral bone osteoblast exosomes, has been shown to activate the catabolic gene expression in articular cartilage chondrocytes by RNA sequencing and miRNA profiling. Yang et al. reported that the interaction between vascular endothelial cells and chondrocytes showed that exosomes derived from vascular endothelial cells decreased the ability of chondrocytes to resist oxidative stress by inhibiting autophagy and p21 expression, which raised the amount of the ROS in the cells and induced apoptosis [51]. Liu et al. reported an elevation of circulating exosomal osteoclast-derived microRNAs (OC-miRNAs), which could suppress tissue inhibitors of metalloproteinase-2 (TIMP-2) and TIMP-3 of chondrocytes in the surgery-induced OA model mice [52]. Blocking OC-miRNAs by Cre-mediated excision or short interfering RNA substantially delayed the development of OA in the model mice. Lv et al. found that dysfunctional chondrocyte-derived exosomes inhibited macrophage autophagy and promoted M1 macrophage polarization [53].

### 2.3. Exosomes as Messengers in Cartilage Diseases

Exosomes reveal a novel mechanism that pathogenic signals are communicated among different types of cells in the OA and other disease joints. Liu et al. reported that internalization of inflammatory fibroblast-like synoviocyte-derived exosomes can improve macrophage M1 polarization, which in effect promotes co-cultured chondrocytes to exhibit an OA-like phenotype [54]. Intra-articular injection of the particular exosome induces accelerates the development of OA in mice model. According to Kong et al., exosomes from fibroblast-like synoviocytes of OA promote cartilage ferroptosis and damage via delivering microRNA-19b-3p to target SLC7A11 [55]. Wang et al. investigated the impact of T-2 toxin, which is the risk factor for Kashin–Beck disease (KBD), in the development of cartilage injury in KBD. The induction of differential expression of chondrocyte exosomal miRNAs, such as miR-181a-5p, miR-21–3p, miR-152–3p and miR-186–5p, led to a collective regulation of RALA, REL, and MAPK10 genes, ultimately mediating the Ras signaling pathway. These changes eventually disrupted chondrocyte extracellular matrix metabolism, resulting in chondrocyte injury [56]. The pathophysiology of idiopathic short stature (ISS) was examined by Yuan et al., who discovered that higher levels of ISSRL, a particular long non-coding RNA (lncRNA), are a differentiating feature in ISS with high specificity and sensitivity [57]. To put it briefly, exosomes play a crucial role in facilitating communication between chondrocytes and the surrounding tissues.

## 3. Exosome Isolation Methods

**Figure 2:**
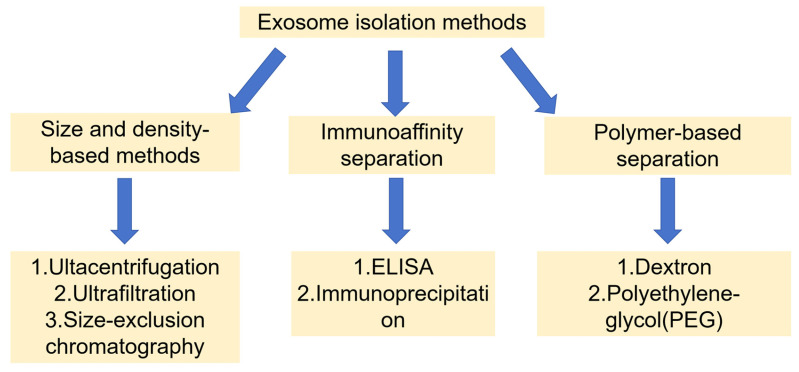
Different isolation methods for exosomes.

The isolation methods of exosomes from different sources are mainly based on three biophysical properties, size and density, immunoaffinity separation, and polymerization precipitation. Numerous investigations have evaluated the yield and purity of exosomes from various isolation methods [57,58,59,60]. Figure 2 illustrates the different isolation techniques for exosomes. Even with recent advancements, conventional methods for isolating exosomes are still challenging. There are several techniques available today for isolating exosomes, and this page outlines the benefits and drawbacks of each technique (Table 2).

The gold standard method for isolating exosomes is ultracentrifugation, in which a sample is centrifuged at a speed of 100,000× *g* [61]. Centrifugation time, rotor type, and centrifugal force all have an impact on its yield and purity. The advantages of ultracentrifugation are large amount of separation, high purity, and reliable separation, while the disadvantages are that it is time-consuming and has a high establishment cost [62]. Ultrafiltration, on the other hand, separates exosomes of certain sizes using membranes with varying molecular weights. The yield using ultrafiltration owned significantly lower processing time and ease of set-up comparable to ultracentrifugation [63]. Size-exclusion chromatography uses a single column to serially elute extracellular vesicle isoforms of different sizes. Larger particles discharged through the mobile phase while the smaller particles attached to pores in the static phase [64]. Unlike the other two methods mentioned earlier, immunoaffinity separation has the ability to isolate exosomes precisely. For instance, the preferred subtypes of extracellular vesicles can be separated and identified from the samples based on the surface marker proteins, such as CD81, CD63, Rab5, CD9, and CD82 [65,66]. The two underlying principles of this technique are the enzyme-linked immunosorbent assay (ELISA) and immunoprecipitation. As for immunoprecipitation, antibodies are immobilized on a solid matrix, typically with the use of polymers or magnetic beads. Surface immunoanalysis is made possible by increased sensitivity and efficiency [67]. Meanwhile, exosomes can be precipitated more easily by overnight incubation with polyethylene glycol (PEG) than by low-speed centrifugation [68]. Another innovative precipitation method is the aqueous two-phase system, which separates particles into various phases to produce more exosomes. When it is treated with two different solutions, dextran and PEG solution, a lower phase accumulated exosomes and an upper phase accumulated proteins and other molecules [69,70].

Overall, the method of isolation and purification can affect the purity and quality of exosomes, and it is important to ensure consistent and high-quality exosomes using standardized and validated methods. Each method has its advantages and disadvantages, and the selection of an appropriate isolation method can optimize the yield and purity of exosomes.

## 4. Exosomes as a Tool for Diagnosis of OA

Most exosomes in the OA microenvironment are known to have “negative” effects on joints. The assessment of exosome content can be used as a basis for the staging diagnosis of OA [71]. Exosomes may be useful as diagnostic tools because of the distinct expression of exosomes in synovial fluid, which is made by synovial fibroblasts and chondrocytes, between healthy and abnormal joints. Theoretically, the up-regulated/down-regulated exosomal miRNAs involved in OA pathogenesis may serve as OA biomarkers. The protein profiles of SF-derived exosomes in individuals with OA, gout, axial spondyloarthritis, and rheumatoid arthritis (RA) were reported by Hunang et al. The result showed that the exosome-derived PZP level of SF in RA was higher than that in OA [44]. Exosomes were isolated from osteoarthritis patients’ serum and synovial fluid, and their miRNA expression was examined by Xie et al. The results demonstrated that blood-derived exosomes are not a perfect representation of synovial fluid exosomes, since 31 upregulated and 33 downregulated miRNAs were detected in synovial fluid as opposed to serum. The properties of exosomes extracted from synovial fluid and their function in osteoclast differentiation in several forms of inflammatory arthritis (OA, RA, gout, and AS) were also examined by Song et al. [72]. OA patients had the lowest levels of synovial exosomes. As a result, osteoclastogenesis was significantly higher in the macrophages that treated with RA patients’ exosomes, compared to those treated with OA patients’ exosomes. Xue et al. investigated the biological functions and potential clinical importance of long non-coding RNAs (lncRNAs) and mRNAs from serum exosomes. There is a combination of three DE-mRNAs, CCL5, MPIG6B, and PFKP, had a good potential for differentiating RA from OA [73]. The impact of exosome-encapsulated miR-127-3p from bone marrow-derived mesenchymal stem cells (BM-MSCs) on OA was examined by Dong et al. They discovered that exosomal miR-127-3p from BM-MSCs inhibits CDH11 in chondrocytes, preventing the activation of the Wnt/β-catenin pathway and reducing chondrocyte damage in OA [26].

The patient’s joint condition can be evaluated individually and thoroughly, as the mechanism of exosome involvement in OA is gradually understood and enough information is gathered from the “database.” Based on the findings of exosome, the necessary materials could be put together into “therapeutic” exosomes to provide individualized treatment and optimize the therapeutic effect.

## 5. The Therapeutic Potential of Exosomes Derived from Natural Cells for OA

**Figure 3:**
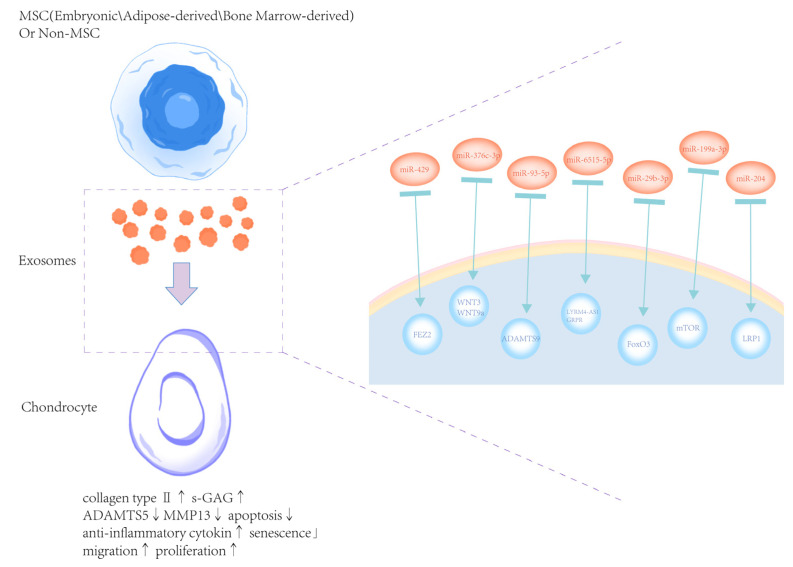
The therapeutic potential of exosomes derived from natural cells for OA [75,76,77,78,79,80,81]. Exosomes secreted by mesenchymal stem cells (MSCs) or non-MSCs deliver functional miRNAs to chondrocytes, regulating various target genes and signaling pathways. These effects promote type II collagen and s-GAG expression, suppress cartilage degradation enzymes (ADAMTS5, MMP13), inhibit apoptosis, and reduce inflammatory cytokines and senescence. Simultaneously, they enhance chondrocyte migration and proliferation (↑ indicates upregulation/increase; ↓ indicates downregulation/decrease), contributing to cartilage repair and OA symptom relief.

Exosome-based OA treatment has drawn increasing interest. Exosomes released by MSCs function as intercellular messengers that deliver bioactive substances, such as proteins, lipids, mRNA, and miRNA, which can be used to treat OA in a number of ways [74]. Exosomes produced from non-mesenchymal stem cells have also progressively emerged as a novel research topic (Figure 3).

### 5.1. Exosomes from Embryonic MSCs

Following medial meniscus instability surgery on the knee joint, Wang et al. demonstrated that intra-articular injection of exosomes derived from embryonic MSCs reduced cartilage destruction and matrix degradation in an OA model. Additional in vitro research revealed that these exosomes preserved the chondrocyte phenotype by promoting collagen II synthesis and inhibited the expression of ADAMTS5, which is a typical matrix-degrading enzyme. Additionally, immunocytochemistry validated the exosomes and collagen II positive chondrocytes colocalized [82].

### 5.2. Exosomes from Adipose-Derived MSCs

Liao et al. examined the impact of transcriptional factors on cartilage engineering, neocartilage formation, and tissue function during and after chondrogenesis using Infrapatellar fat pad-derived mesenchymal stem cells (IPFP-MSCs). They discovered improvements in enhancement strategies for IPFP-MSC chondrogenic differentiation, including the effects of transcriptional factors, the modulation of released exosomes, MSC delivery mechanisms, and ethical and regulatory points concerning the development of MSC products [83]. According to Meng et al.’s investigation on the function of adipose tissue-derived stromal cell (ADSC), ADSC exosomes could delay OA development and may be internalized by chondrocytes to stimulate chondrocyte proliferation via miR-429. Through its promotion of autophagy and targeting of FEZ2, miR-429 reduced cartilage damage in OA [75]. Li et al. investigated the role and mechanism of adipose mesenchymal stem cell (ADSC)-derived exosomes (Exos) in OA-induced chondrocyte degradation and synovial hyperplasia, thus improving the quality of life of patients. They found that the miR-376c-3p in hADSC-derived Exos mitigated OA-induced chondrocyte degradation and synovial fibrosis. MiR-376c-3p in hADSC-derived Exos repressed the WNT-beta-catenin pathway by WNT3 or WNT9a, and then mitigating OA-induced chondrocyte degradation and synovial fibrosis, thereby providing a theoretical basis for clinical implementation of treatment [76]. Li et al. reported that exosomal miR-93-5p from adipose-derived MSCs could reduce the release of inflammatory factors through the ADAMTS9 signal axis in arthritic mice cell models [77].

### 5.3. Bone Marrow MSC-Derived Exosomes

Jiang et al. found that exosomes produced from BM-MSCs could suppress cell apoptosis, promote cell proliferation, and control c-MYC to control the amount of glutamine metabolism in chondrocytes [84]. Zhou et al. discovered that BM-MSC-derived exosomes may considerably improve chondrocyte cell viability in response to IL-1β therapy [85]. Chondrocytes’ expression levels of the pro-inflammatory genes IL-1β, IL-6, NF-κB, TNF-α and TNF-β of chondrocytes significantly reduced after co-culture with exosomes. Li et al. found that BM-MSC-derived exosomes inhibited mitochondrial membrane damage, ROS production, and the protein expression of PINK1 and Parkin [86]. In the synovium of OA rats, BM-MSC-derived exosomes alleviated cartilage damage, suppressed M1 polarization, and promoted M2 polarization. When these specific exosomes were treated to OA rats, the expression of PINK1 and Parkin in the synovium and the serum levels of IL-6, IL-1β, and TNF-α decreased, while the level of IL-10 increased. Jin et al. found that BM-MSC-derived exosomes alleviated subchondral bone remodeling and cartilage degradation in OA rat models [87]. Administration of BM-MSC-derived exosomes could reduce joint damage and restore the trabecular bone volume fraction, trabecular number and connectivity density of OA rats. In addition, in vitro assays demonstrated that BM-MSC-exosomes could preserve the chondrocyte phenotype by promoting the expression of collagen type II and inhibiting IL-1β-induced senescence and apoptosis. Additionally, exosomal lncRNA MEG-3 decreased IL-1β-induced chondrocyte senescence and apoptosis, that lncRNA MEG-3 may contribute to the anti-OA properties of BM-MSC exosomes. Furthermore, exosomal lncRNA MEG-3 also reduced the senescence and apoptosis of chondrocytes induced by IL-1β, suggesting that lncRNA MEG-3 may contribute to the anti-OA effects of BM-MSC exosomes. Wang et al. demonstrated that BM-MSC-derived exosomes reversed the effects of IL-1β, which drastically reduced chondrocyte viability and promoted apoptosis [78]. Xia et al. observed that chondrocytes can internalize miR-125a-5p, and exosomes produced from BMSCs were abundant in this protein [88]. Alongside the down-regulation of MMP-13 and the up- regulation of Collagen II, aggrecan, and SOX9 in vitro, miR-125a-5p has the ability to accelerate chondrocyte migration. Dong et al. investigated the role of BMSC-derived exosomes in quercetin-mediated progression of OA, and found that exosomes significantly attenuated OA progression through the upregulation of miR-124-3p [89]. Shen et al. examined the mechanism of BMSC-derived lncRNA TUC339 on OA and discovered that BMSC-derived exosomes improve OA by increasing TUC339 expression to promote M2 polarization, suppressing inflammation, and promoting chondrocyte activity, which provides a solid foundation for future transplantation therapy of MSCs for OA [90]. Cheng et al. discovered that IL-1β stimulation reduced cell viability, raised Fe^2+^/ROS/MDA levels, decreased GSH levels, and increased the frequency of TUNEL-positive cells and the level of ACSL4 in rat chondrocytes. These effects were countered by exosomes produced from BMSCs [91]. By suppressing METTL3 and downstream m6A alteration of ACSL4 mRNA, BMSC-derived exosomes prevented chondrocyte ferroptosis.

### 5.4. Exosomes from Human Umbilical Cord-Derived MSCs (hUC-MSCs-Exos)

hUC-MSCs-Exos demonstrated the capacity to deliver lncRNA H19 to chondrocytes in the Yan et al. research [79]. Mechanistically, exosomal lncRNA H19 potentiated osteochondral activity by acting as a competing endogenous sponge of miR-29b-3p, which directly targeted FoxO3. Yang et al. discovered that hUC-MSCs-Exos markedly increased the expression of collagen II and decreased the expression of MMP-13 and ADAMTS-5 in chondrocytes stimulated by IL-1β [92]. According to Wang et al., hUC-MSCs-Exos showed the opposite impact of IL-1β on chondrocytes in terms of MMP13 and collagen type II alpha 1 (COL2A1) [93]. Li et al. investigated the potential of hUC-MSCs-Exos in alleviating OA, and found that the high efficacy of hUC-MSCs-Exos in promoting chondrocyte proliferation and migration and also inhibiting chondrocyte apoptosis. Furthermore, hUC-MSCs-Exos could control macrophage polarization in vitro and reverse chondrocyte damage induced by IL-1β [94].

### 5.5. Exosomes from Non-MSCs

As exosomes are widely present in the human body, the non-mesenchymal stem cell-derived exosomes, which have a rich variety and quantity reserve, play a role in the treatment of osteoarthritis. In vitro, Xu et al. reported that platelet-derived exosomes promoted chondrocyte proliferation and migration [95]. In total, 1797 genes exhibited differential expression in chondrocytes following treatment with platelet-derived exosomes. In vivo, Fu et al. discovered that exosomes generated from dental pulp stem cells (DPSC) efficiently reduced cartilage degradation and synovial inflammation, prevented the development of osteophytes and bone sclerosis, and enhanced abnormal subchondral bone remodeling [96]. Additionally, they discovered that by preventing TRPV4 activation, exosomes generated from DPSCs suppressed osteoclast activation in vivo. According to Zhao et al., exosomes produced from subcutaneous fat stromal cells may reduce the degree of pathological severity of cartilage through miR-199a-3p, which may modulate the mTOR-autophagy pathway in the rat model of OA [80]. Certain serum exosome subpopulations demonstrated distinct associations with knee OA (KOA) pain and functional limitations, according to Mustonen et al. They investigated the relationships between these exosome surface markers and articular cartilage degradation, subjectively and objectively measured pain, and functional limitations in primary KOA and reported several significant predictors of small exosomes, such as CD41, CD63 and CD9 [97]. According to Xu et al., exosomes produced by osteocytes prevented ECM deposition and death while promoting cell survival and migration [98]. Additionally, the DLX2 expression was elevated by exosomes, and the Wnt pathway was stimulated by a DLX2 knockdown. As a result, transmitting DLX2 by osteocytes exosomes could reduce OA in mice, which presents a new therapeutic target.

### 5.6. Exosomes from Plant

Exosomes generated from plants have become more and more popular in recent years for their diverse origins and low immunogenicity. Another benefit is that, in contrast to mammals, plants do not harbor human or zoonotic infections. Exosomes generated from plants have been shown in several studies to be promising candidates for development as nanocarriers into delivery platforms. Plant-derived exosomes contain natural components that have shown anti-tumor, anti-inflammatory, and tissue-regenerative qualities in addition to being designed exosomes for targeted delivery systems [99]. For the first time, it was discovered that plant-derived mitochondrial DNA (mtDNA) is transferred from plants to mammals. It was also shown that mtDNA is internalized into macrophages through vesicles and facilitates the conversion of tumor macrophages into an anti-tumor phenotype.

### 5.7. Exosomes from Food-Derived Sources

In addition to traditional mammalian and plant-derived EVs, food-derived EVs have emerged as a novel and promising class of bioactive agents for OA intervention. These exosome-like nanoparticles have been successfully isolated from edible sources such as bovine milk, ginger, grapes, and broccoli, and are characterized by favorable properties including biocompatibility, oral availability, and low immunogenicity [100]. Among them, bovine milk-derived EVs have shown the most consistent evidence in osteoarthritis-related studies. Pieters et al. reported that bovine milk-derived EVs significantly attenuate sulfated glycosaminoglycan loss and suppress the expression of cartilage-degrading enzymes such as ADAMTS5 and MMPs in human OA cartilage explants. Mechanistically, these effects are partially mediated by the delivery of TGF-β and miR-148a, which contribute to chondrocyte homeostasis [101]. In the destabilization-of-medial-meniscus mouse model of OA, oral administration of bovine milk-derived EVs was shown to attenuate cartilage degeneration by increasing hyaline cartilage thickness, reducing OARSI scores, enhancing extracellular matrix synthesis, and restoring disrupted gut microbiota composition, suggesting a gut–joint axis-mediated therapeutic mechanism [102]. Moreover, bovine milk-derived EVs have been engineered as carriers for small RNAs (such as miRNAs) and therapeutic agents, facilitating cartilage protection and extracellular matrix remodeling, and offering a scalable, biocompatible, and non-invasive drug delivery platform [103,104]. Ong et al. emphasized the nutritional and regulatory roles of milk EVs in systemic inflammation, metabolic balance, and extracellular matrix turnover, all of which are key factors in OA pathogenesis and progression [100]. Overall, food-derived EVs, particularly those from bovine milk, provide a promising, biocompatible, and mechanistically diverse approach for osteoarthritis therapy, although further clinical studies are needed to establish their efficacy.

### 5.8. Exosomes from Bacterial-Derived Sources

Bacterial extracellular vesicles (BEVs) are nanoscale membrane-bound structures secreted by both commensal and pathogenic bacteria, and have recently been recognized as key mediators of host–microbe communication and immune modulation [105,106]. Niu et al. proposed that BEVs may influence OA progression through the gut–joint axis by affecting intestinal barrier integrity, microbial composition, and systemic immune responses [107]. Although direct experimental evidence is currently limited, BEVs have been implicated in modulating key inflammatory pathways, such as TLR4/NF-κB signaling and in regulating the balance between Th17 and Treg cells, which may contribute to OA-related immune alterations. Given their ability to carry immunologically active molecules and interact with host epithelial and immune cells, BEVs represent a promising but underexplored avenue for microbiota-targeted interventions in OA. Further studies are needed to clarify their mechanisms and therapeutic potential.

## 6. Comparison of Therapeutic Potential of Exosomes Between Different Sources

Yin et al. reported that exosomes of infrapatellar fat pad-derived stem cells significantly inhibit the degradation of cartilage extracellular matrix, compared to the exosomes of subcutaneous adipose tissue-derived stem cells [108]. Zhu et al. examined the impact of exosomes produced by induced pluripotent stem cell-derived MSCs (iMSC-Exos) and synovial membrane MSCs (SMMSC-Exos) on the management of osteoarthritis. After the injection into the joint to the OA mice model, iMSC-Exos and SMMSC-Exos both attenuated OA, promoted chondrocyte migration and proliferation, but iMSC-Exos has shown a stronger therapeutic effect [109]. Compared to BMSC-Exos, Zhang et al. discovered that IL-1β-treated chondrocytes treated with dECM (decellularized extracellular matrix)-pretreated BMSC-Exos exhibited improved anabolism, migration, proliferation, and anti-apoptosis characteristics. By upregulating miR-3473b, which targets PTEN, they discovered that dECM-BMSC-Exos can delay the progression of OA by promoting migration, enhancing anabolism, and preventing the apoptosis of chondrocyte [110].

The studies mentioned above demonstrate that the therapeutic impact of exosomes derived from different origins varies. The best cell source for exosomes has yet to be found, and the mechanism underlying the variations in therapeutic impact must be investigated.

## 7. Exosome-Based Biochemical Engineering Strategy on OA

**Figure 4:**
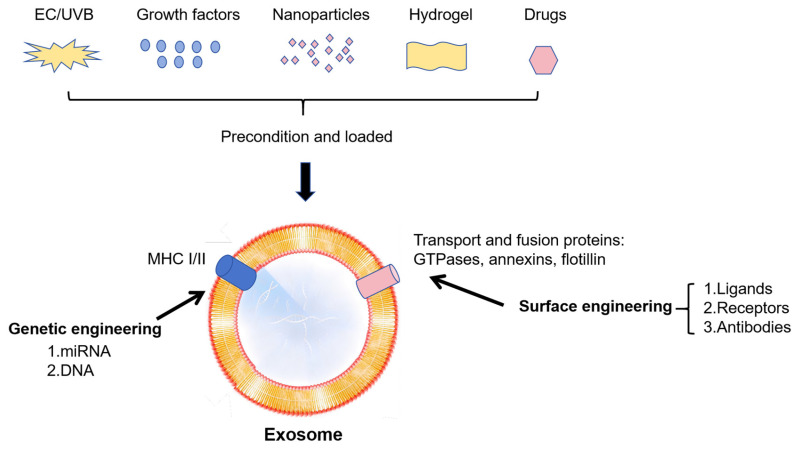
Engineered exosomes for cartilage repair, including surface and genetic engineering, precondition and loaded. Exosomes can be modified through genetic engineering (e.g., miRNA or DNA manipulation) or surface engineering (e.g., ligands, receptors, antibodies), as well as preconditioning or loading with external stimuli such as UVB, growth factors, nanoparticles, hydrogels, or drugs. These strategies enhance the bioactivity, targeting, and therapeutic potential of exosomes in osteoarthritis treatment.

Exosomes’ capacity to control cellular functions, including inflammation, proliferation, and differentiation, makes them promising drugs for the treatment of OA. However, some defects, including poor targeting, short half-life in vivo, and low bioavailability, may restrict their effectiveness. Researchers are investigating the use of modified exosomes to enhance treatment outcomes in OA in order to overcome these constraints. Here is a summary of several strategies for maximizing exosome quality (Figure 4 and Table 3).

### 7.1. Advantages of Exosomes as Advanced Drug Delivery

Exosomes have been extensively evaluated for their crucial role as intercellular communication vehicles [125]. In addition, they have the potential for clinical application as advanced drug delivery and therapeutics. Recent studies have highlighted the broad applicability of engineered exosomes in the treatment of specific diseases, and their clinical applications continue to expand (Table 4).

Furthermore, exosomes are generally not recognized as foreign by the immune system and are therefore less likely to be eliminated. The immunogenicity of exosomes may be related to their surface adhesion proteins and carrier ligands. However, it is uncertain if exosomes lack major histocompatibility complex components. Multiple intravenous infusions of MSC-derived exosomes over a 2-week period were likewise well-tolerated and did not result in negative side effects in a patient with graft versus host disease [126]. Conversely, xenotransplanting human embryonic MSCs into immunocompetent mice to repair cartilage caused an adverse tissue reaction immediately, which led to inadequate reparative outcomes [127].

### 7.2. Strategy on Cells

Exosomes have drawn a lot of interest in recent years due to their drug loading and signal-carrying capacity. Modification of exosome-derived cells for better OA treatment has been attempted lately, exhibiting enormous potential as future therapeutic strategies for OA.

Exosomes from hypoxia-cultured human adipose stem cells have been shown to reduce articular chondrocyte inflammation and the development of post-traumatic OA by Chang et al. [128]. Additionally, seven potential miRNAs were discovered in these exosomes by NGS and bioinformatic analysis, which might help control oxidative stress in cells and attenuating cell senescence. Lou et al. found that exosomes derived from fucoidan-directed induction mesenchymal stem cells, which enriched in MiR-146b-5p, could inhibit TRAF6 and downstream inflammatory responses and extracellular matrix degradation. Meanwhile these exosomes promoted chondrocyte autophagy for the protection of osteoarthritic cartilage cells [129]. Sun et al. discovered that exosomes produced from synovial mesenchymal stem cells modified by bone morphogenetic proteins-7 (BMP-7) enhanced the proliferation of chondrocytes and macrophages caused by LPS and showed a greater capacity to alleviate inflammation by enhancing the M2 polarization of macrophages [112]. Mao et al. demonstrated that treatment with exosomes derived from human bone marrow MSCs overexpressing miR-92a-3p (MSC-miR-92a-3p-Exos) increased the expression of matrix genes in PHCs and cartilage proliferation in MSCs, respectively. On the other hand, by increasing WNT5A expression, MSC-anti-miR-92a-3p-Exos therapy inhibited chondrogenic differentiation and decreased cartilage matrix synthesis. Therefore, exosomal miR-92a-3p shows the function of Wnt pathway inhibition and its potential as a disease-modifying osteoarthritis drug [128].

Tao et al. demonstrated that exosomes derived from miR-140-5p-overexpressing synovial MSCs (SMSC-140-Exos), which were transfected with miR-140-5p, enhanced the proliferation and migration of articular chondrocytes without damaging extracellular matrix secretion in vitro, while SMSC-140-Exos successfully prevented OA in vivo [129]. Sun et al. evaluated the expression of miRNAs in exosomes produced from human bone MSCs (hBMSCs) with and without chondrogenic stimulation, they found that 106 miRNAs were downregulated, including miR-377-3p, and 35 upregulated, including miR-1246, miR-1290, miR-193a-5p, miR-320c, and miR-92a [91]. Furthermore, they extracted exosomes enriched in miR-320c and validated that these specific exosomes were more efficient in promoting osteoarthritis chondrocyte proliferation, down-regulated MMP13 during hBMSC chondrogenic differentiation, compared to the normal exosomes derived from hBMSCs. These studies indicated that overexpression of the key signaling molecules in exosomes may be a good strategy.

Wang et al. investigated the molecular mechanism of TGF-β1 in regulating chondrocyte proliferation through MSC-exosomes [130]. They discovered that miR-135b expression in MSC-exosomes was elevated by TGF-β1 stimulation, and that miR-135b produced from MSC-exosomes improved the viability of chondrogenic cell line. Furthermore, via targeting Sp1’s 3’UTR, miR-135b suppressed Sp1 expression, which has been shown to have a detrimental impact on chondrocyte proliferation [111]. Thus, Wang et al. demonstrated that TGF-β1 induced MSC-exosomes, enriched in miR-135b, could stimulate cartilage regeneration by promoting chondrocyte proliferation. Similarly, Shao et al. compared the effects of exosomes derived from BMSCs (Exo^BMSC^) and exosomes derived from parathyroid hormone (PTH)-induced BMSCs (Exo^PTH^) in the treatment of OA [131]. Both Exo^BMSC^ and Exo^PTH^ reduced the expression of IL-2, TNF-α, and IL-6 in chondrocytes, while Exo^PTH^ exhibited stronger anti-inflammatory, proliferation, migration, and production of the extracellular matrix compared to Exo^BMSC^. According to Kim et al., osteoarthritic SW982 cells (the human synovial cell line SW982 treated with IL-1β and TNF) show markedly increased anti-inflammatory activity when exposed to BM-MSC exosomes primed with IL-1β [23]. The NF-κB pathway is inhibited by IL-1β-primed MSC exosomes, which is mediated by miRNAs such as miR-147b. The results above indicated that cytokines induced exosomes may achieve better therapeutic effects for OA.

Mechanical stimulation provides a novel strategy for optimizing the exosomes. Xu et al. found pulsed electromagnetic field (PEMF)-exposed MSC-derived exosomes could be endocytosed by osteoarthritis chondrocytes. The PEMF-exposed AMSC-derived exosomes significantly reduced the extracellular matrix degradation and inflammation of IL-1β-induced chondrocytes in comparison to the AMSC-derived exosomes alone [132]. The exosomes treated with 75 Hz PEMF presented a more significant inhibiting impact than those with 15 Hz and 45 Hz PEMFs.

### 7.3. Strategy on 3D Culture

In a multicellular organism, the extracellular matrix envelops the highly ordered, three-dimensional (3D) tissue cells [133]. In comparison to the 2D culture, Han et al. found that the optimized 3D culture offered an ideal environment for the proliferation of human synovial cells and the release of exosomes. When it came to mending cartilage defects, the 3D-cultured exosomes showed better healing capabilities than those obtained from the 2D culture and showed more potential for raising chondrogenic gene expression in vitro.

### 7.4. Strategy on Deficiencies of Exosomes

Numerous investigations have discovered that modifying the intrinsic properties of exosomes can increase therapy effectiveness. GuiLu-ErXian Glue, a herbal treatment that is often used in traditional Chinese medicine, was discovered to be able to delay the aging process in the MSC senescence process and possibly increase MSC-induced chondrogenesis through the release of exosomes in vitro. By using lipid insertion to modify the exosome surface with the cartilage affinity peptide (CAP), Zhang et al. created chondrocyte-targeting exosomes called CAP-Exo. These exosomes were then loaded with siRNA against MMP13 (siMMP13) inside, creating CAP-Exo/siMMP13. In a rat OA model, intra-articular delivery of CAP-Exo/siMMP13 raised COL2A1 and proteoglycan in cartilage, while decreasing MMP13 levels [116]. In the study by Zhang et al., buffer pH is used as a charge-reversal switch to create charge-reversed cationic exosomes. Due to weak-reversible ionic binding with aggrecan-glycosaminoglycan, cationic exosomes were able to pass through the full thickness of early-stage arthritic human cartilage and effectively deliver the encapsulated eGFP mRNA to chondrocytes located in tissue deep layers, whereas unmodified anionic exosomes do not [117]. To enhance the targeting capability, Xu et al. discovered combining an MSC-binding peptide E7 with the exosomal membrane protein Lamp 2b yields exosomes with E7 peptide displayed on the surface (E7-Exo), which has the potential to target synovial fluid-derived mesenchymal stem cells (SF-MSC) [134]. Then, the E7-Exo could deliver Kartogenin (KGN), a small molecule that has been discovered to induce a differentiation of SF-MSCs to chondrocytes, efficiently penetrated SF-MSCs and induce higher degree of cartilage differentiation, compared to KGN alone or KGN delivered by exosomes without E7.

### 7.5. Strategy on Exosomes Carrier

In the field of OA therapy, hydrogel materials have been widely used as cell or factor scaffolds to better fill the cartilage defect and provide a mode for cartilage regeneration, due to their unique features, such as high water content, biocompatibility, swelling behavior and modulated 3D networks [135]. Thus, there are a lot of prospects for using hydrogel materials and exosomes as an OA treatment approach.

Cao et al. synthesized exosomes on membranes using chondrocyte-targeting polymers and encapsulated them in thiolated hyaluronic acid microgels, which form a “two-phase” releasing system, and synergistically facilitated the repair of OA cartilage in a rat model [118]. In Wan et al.’s study, novel photocrosslinking spherical gelatin methacryloyl hydrogel (GelMA)-encapsulated cartilage affinity WYRGRL (W) peptide-modified engineered exosomes were developed for OA treatment. These exosomes demonstrated a strong action promoting anabolism and suppressing catabolism in vitro, as well as an effective targeting effect on chondrocytes [119]. In order to build an injectable microgel system, Chen et al. used microfluidics and photopolymerization to encapsulate chondrocyte-affinity peptide-incorporated hybrid exosomes, which loaded with an FGF18-targeted gene-editing tool in methacrylic anhydride-modified hyaluronic hydrogel microspheres. This system has the capacity to synergistically promote cartilage regeneration, decrease inflammation, and prevent ECM degradation both in vitro and in vivo [120]. A hydrogel microsphere containing SOD3-enriched exosomes (S-EXOs) was created by Cao et al. Purified S-EXOs preserved the integrity of extracellular matrix metabolism and increased chondrocytes’ antioxidant capability. Hydrogel microspheres containing S-EXO could efficiently deliver SOD3 to cartilage and significantly delay the development of OA [121]. Ma et al. used Atf5-modRNA in conjunction with BMSC-derived modified exosomes to exert cytoprotective effects on chondrocytes in articular cartilage. In order to produce a prolonged release of exosomes, they then used an injectable thermosensitive hydrogel (PLGA-PEG-PLGA) as a carrier, suggesting a very successful approach to treating OA [122].

Another crucial technique for hydrogel modification is nanofiber reinforcement, which has superior mechanical qualities, porosity, biodegradability, and controlled release design potential. Like extracellular matrix (ECM), nanofibers may regenerate to support cell growth, adhesion, and proliferation [136]. In order to facilitate the healing of cranial defects in a rat model, Lu et al. created GelMA-HAMA/nHAP nanocomposite hydrogels loaded with exosomes, which generated from human urine stem cells. These hydrogels demonstrated good controlled release qualities and suitable mechanical properties. An injectable hydrogel containing exosomes that is very sticky and was inspired by mussels was investigated by Zhang et al. [137]. A crosslinked network of chondroitin sulfate, alginate-dopamine, and regenerated silk fibroin (AD/CS/RSF) was used to create the hydrogel, which has a strong attachment to the wet surface. In addition to recruiting BMSC migration and inflation, the AD/CS/RSF/EXO hydrogel with encapsulated exosomes encouraged BMSC proliferation and differentiation, and eventually sped up extracellular matrix remodeling following injection in cartilage defect model.

### 7.6. Strategy on 3D Printing

Three-dimensional printing technology allows better tuning of composite scaffolds in terms of shape, size, and porosity for in vivo transplantation. In a recent study, MSC-derived exosomes were composited with collagen/chitosan by 3D printing technology (3D-CC-BMExos) with good mechanical properties and biocompatibility, and scaffolded with a porous network structure. Their high porosity promoted cell adhesion with significantly higher cumulative exosome release, compared to the non-3D printed scaffold [138]. Li et al. created a new bioinspired double-network hydrogel scaffold using exosomes obtained from hADSCs and tissue-specific decellularized extracellular matrix with 3D printed [139]. In vitro, 3D-printed microenvironment-specific heterogeneous bilayer scaffolds facilitate the attachment, migration, proliferation, and chondrogenic and osteogenic differentiation of BMSCs, simultaneously it effectively speed up the concurrent regeneration of cartilage and subchondral bone tissues in vivo.

Thus, the advancement of 3D printing technology offers a fresh approach for the application of exosomes in OA treatment. In order to improve the therapeutic benefits of exosomes and create a more optimal geometric structure, scaffolds with expected effects can be constructed using 3D printing technology, such as stereolithography [113,140]. In the work by Abdollahiyan et al., chondrocytes were loaded with a photo-crosslinkable bioink that contained varying amounts of silk methacrylate (SilMA) and polyethylene glycol diacrylate (PEGDA), in order to biofabricate 3D-bioprinted cartilage constructions [123]. In addition to achieving the most dependable rheological characteristics, printability, and mechanical and degradation qualities appropriate for cartilage regeneration, the printed SilMA-PEGDA hydrogel structures also had the proper internal porous structure. For 3D printing multiscale scaffolds that integrate the micro and macro environments of the natural articular cartilage, Yin et al. developed a modular hydrogel-based bioink with chondrocytes embedded in microspheres [124]. For creating the multiscale hydrogel-based scaffolds, the gel-in-gel 3D bioprinting technique might be used to create the modular hydrogel bioink, in which the cultured cells demonstrated excellent differentiation and proliferation.

## 8. Future Directions

### 8.1. Local Sustained Release System Enables the Efficacy of Agents at Small Dosages

Currently, the majority of research in animal models and preclinical trials indicates that exosome management is crucial for cartilage/OA healing. The issue is that the repeated administration, in order to maintain effective concentration, may increase the risk of pain and side effects in the injection site. As offering a controlled release platform of exosomes, the approach of developing bio-scaffolds as “exosome carriers” has been regarded as a solution [113]. In addition, exosomes formulated with biocompatible and biodegradable hydrogels can be delivered to the target site or nearby locations to promote the concentrations of therapeutic molecules. There are two benefits of administering local joint lesions under control. One is to prolong the drug’s duration at a low dose by controlling the drug’s degradation and maintaining its effective concentration. The second is that it is beneficial to utilize “exosome carriers” with varying sustained release effects based on the extent of cartilage destruction, which is crucial for future individualized therapy of OA.

### 8.2. Drug Loading Techniques for Modular Design of Contents

As previously stated, exosome biogenesis processed by protein-mediated budding, which encapsulates and secretes internalized material, including nucleic acids and proteins. The technologies of exosome drug loading can be divided into two categories. One method is to inject drug-loaded exosomes into donor cells by transfection or co-incubation. Another way is to load the medications, which released outside the donor cells, into exosomes by direct mixing, electroporation, sonication, saponin treatment, extrusion, and freeze/thaw cycles. As previously stated, RNA transfection is now the most widely utilized approach for increasing the effectiveness of exosomes in OA. As a result, it is critical to investigate the loading process of exosomes and develop therapeutic cargo with the highest efficacy, which will be extremely beneficial for OA therapy in the future.

### 8.3. Future Personalized and Precision Treatment Strategies

Individualized precision medicine refers to obtaining a large amount of personalized information from patients, and then developing precise treatment plans based on this information. With remarkable developments in “next generation” diagnostic technology, we have an improved understanding of the causes of various cartilage illnesses. Furthermore, in recent years, the introduction of liquid biopsy technology has made it simpler to access OA databases, such as methylation alterations and exosome information. The core principle of precision medicine is to cure or “ablate” diseased disorders with little harm to normal tissues based on sufficient big data. It could be practical to utilize the methods described above that put together the required cargos, such as DNA, RNA, proteins and medications, into modular exosomes with good targeting capabilities, customizable and optimized therapeutic impact. As a result, modular exosomes will be particularly effective in personalized and precision medicine.

### 8.4. Translational Challenges and Clinical Outlook

Although exosomes show promise for OA therapy, clinical translation faces challenges in large-scale, reproducible manufacturing, and also in the standardization of isolation, purification, and storage [141]. Bioengineering strategies such as surface modification and therapeutic cargo optimization can enhance targeting specificity and functional efficacy, but may also increase complexity in regulatory approval processes. Isolation technologies must strike a balance between yield, purity, and scalability, while regulatory classification, whether as biologics, cell-free therapeutics, or drug delivery devices, has yet to be definitively established [142]. To enable safe and effective clinical application, harmonized international guidelines, validated potency assays, and standardized operational protocols will be indispensable.

## 9. Conclusions

OA is a complicated disease involving a number of elements, including a synovial fluid microenvironment, joint cells and inflammatory factors. Exosomes are known to act as carriers of pathogenic signals and accelerate the progression of OA. The targeting and signal transduction of exosome in the OA microenvironment have been continuously investigated. The analysis of exosome content also has been deep investigated as it may be a novel approach to OA early diagnosis.

Although exosomes have a negative impact on the occurrence and development of OA, they can also play a positive role in the treatment of OA. In recent years, many studies have found that exosomes released by some natural cells, including mesenchymal stem cells and non-mesenchymal stem cells, have therapeutic potential for OA. Specific content and related mechanisms of these therapeutic exosomes have also been research hotspots in recent years.

**Figure 5:**
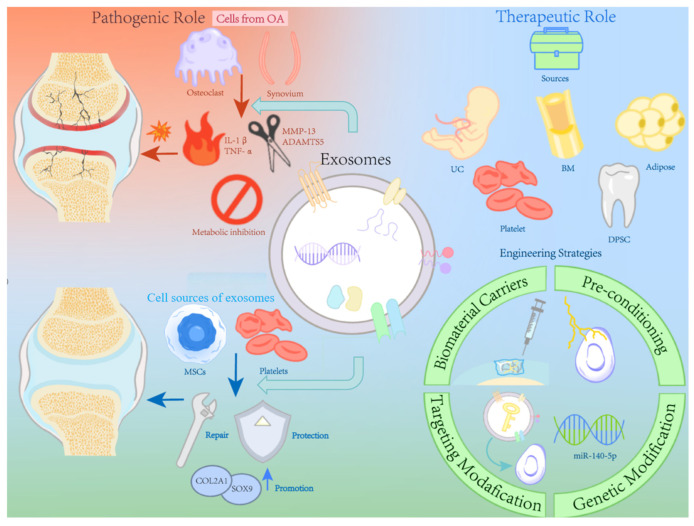
Exosomes in OA: Dual roles in pathogenesis and therapy. This figure provides a comprehensive summary of the dual roles of exosomes in OA. On the pathogenic side, exosomes participate in inflammation, catabolic signaling, and cartilage degradation. On the therapeutic side, they contribute to inhibiting apoptosis, anti-inflammation, and cartilage repair. The lower section illustrates emerging strategies to enhance the therapeutic utility of exosomes, including targeting modification, genetic modification, biomaterial carriers, and pre-conditioning, which collectively aim to improve targeting, promote repair, and protect cartilage from degradation.

The combination of cell tissue engineering and exosomes appears to be a feasible approach to treat OA. At the microscopic level, enhancing exosome characteristics, either directly or indirectly, can increase the effectiveness of OA therapy. At the macro level, an effective scaffold as exosome carrier should be constructed to achieve long-term and sustainable releasing. In particular, the advancement of 3D printing technology contributes in the creation of ideal scaffolds for exosome delivery. A summary illustration of the dual roles and enhancement strategies of exosomes in OA is provided in Figure 5.

Currently, research on the pathophysiology and therapy of OA with exosomes is still in the preclinical stage. The clinical translation of exosome therapy remains challenging. Meanwhile, some important issues still need to be resolved, such as the source, releasing rate, therapeutic dosages and personalized application of exosomes. However, we believe that as biotechnology advances, these problems will be solved in the future.

## Figures and Tables

**Table 1:** The role of exosomal ncRNAs in the pathogenesis of OA.

Kinds of RNAs		Sample	Exosome Source	Role	Reference
miRNAs					
	miRNA-100-5p	Human	Human umbilical cord MSCs	Inhibits cyclic strain-induced ROS production and apoptosis in chondrocytes by targeting NOX4	[19]
	miRNA-126-3p	Rats	Synovial fibroblasts	Promotes chondrocyte proliferation and suppresses apoptosis by constraining chondrocyte inflammation	[20]
	miRNA-206	Mice	Bone marrow-derived MSCs	Promotes proliferation and differentiation of osteoblasts in OA by reducing ELF3	[21]
	miR-136-5p	Human	Bone marrow-derived MSCs	Inhibits chondrocyte degeneration in OA by targeting ELF3	[22]
	miRNA-147b	Human	MSCs treated with IL-1β and TNF-a	Inhibits the inflammatory response of OA SW982 cells	[23]
	miRNA-140-5p	Human	Dental pulp stem cells	Inhibits IL-1β-induced chondrocyte apoptosis	[24]
	miRNA-140	Rats	Dendritic cells	Alleviates OA progression in a rat model	[25]
	miR-127-3p	Rats	Bone marrow-derived MSCs	Alleviates OA by regulating the CDH11-mediated Wnt/β-catenin pathway	[26]
	miRNA-1208	Human	Umbilical cord-derived MSCs	Suppress cartilage ECM degradation via decreasing level of pro-inflammatory factors	[27]
	miRNA-26a-5p	Rats	Bone marrow-derived MSCs	Promotes osteogenic differentiation and inhibit adipogenic differentiation	[28]
	miRNA-129-5p	Human	Synovial MSCs	Relieves IL-1β-induced OA by targeting HMGB1	[29]
	miRNA-361-5p	Human	Bone marrow-derived MSCs	Alleviates OA by targeting DDX20 and NF-κB signaling pathway	[30]
	miRNA-9-5p	Rats	Bone marrow-derived MSCs	Alleviates OA degeneration by targeting SDC1 in an OA rat model	[31]
	miRNA-140-5p	Human	Urine-derived stem cells	Inhibits the progression of KOA by mediating VEGFA	[32]
	miRNA-135b	Rats	Bone marrow-derived MSCs	Attenuates cartilage injury by promoting synovial macrophage M2 polarization by targeting MAPK6	[33]
	miRNA-338-3p	Human	Adipose tissue-derived MSCs	Stimulate cell proliferation and inhibit cell apoptosis	[34]
lncRNAs					
	lncRNA-PCGEM1	Human	Fibroblast-like synoviocytes	Facilitates IL-1β-induced apoptosis and cartilage matrix degradation in chondrocytes by targeting the miR-142-5p/RUNX2 axis	[16]
	lncRNA-NEAT1	Human	Bone marrow-derived MSCs	Activate the proliferation and autophagy of chondrocytes	[35]
	lncRNA-H19	Human	Umbilical cord blood MSCs	Improves pain and central sensitization of advanced OA via miRNA-29a-3p/FOS axis	[36]
	lncRNA-PVT1	Human	C28/I2 cells	Modulates chondrocyte viability, apoptosis, and inflammation responses by miR-93-5p/HMGB1/TLR4/NF-κB pathway	[37]
	lncRNA-H19	Human	Fibroblast-like synoviocytes	Promotes chondrocyte proliferation and migration and inhibits matrix degradation in OA possibly by targeting the miR-106b-5p/TIMP2 axis	[15]
circRNAs					
	circRNA-3503	Human	CircRNA3503-overexpressed synovium MSCs	Alleviates chondrocyte apoptosis and ECM imbalance by acting as sponges of miR181c-3p and let-7b-3p	[38]
	circRNA-0001846	Human	Chondrocyte cell line CHON-001 treated with IL-1β	Modulates IL-1β-induced chondrocyte damage by miR-149-5p/WNT5B axis	[39]
	circRNA-BRWD1	Human	Chondrocyte cell line CHON-001 treated with IL-1β	Promotes OA progression by regulating the miR-1277/TRAF6 axis	[18]
	circRNA-0001236	Human	MSCs	Alleviates cartilage degradation through the miR3677-3p/Sox9 axis	[40]
	circRNA-HIPK3	Human	MSCs	Promotes chondrocyte proliferation and migration and suppresses apoptosis via the miR-124-3p/MYH9 axis	[41]

**Table 2:** Exosome isolation techniques and their comparison.

Isolation Methods	Advantages	Disadvantages	Sample Matrix
UC	Gold standard Simplicity of operator Easily required	Time consuming Decreased in biological activity High requirements for equipment Limited mass production	Cell culture medium Serum Urine
Microfluidic technology	Rapid Save samples and reagents High purity and efficiency	Not suitable for mass generation Methods need to be further standardized	Cell culture medium Serum
Ion exchange	Simplicity of operator High purity	Unknown	Cell culture medium
AF4	High purity High efficiency Identify subset	High requirements for equipment and personnel Limited mass production	Cell culture medium Serum Urine
SEC	High purity Commercial kits available High productivity	Co-separation of proteins with similar diameters Not satisfy the downstream application	Cell culture medium Serum Urine Cerebrospinal fluid
Polymer precipitation	Mass production Simplicity of operator	Decreased purity Protein contamination Expensive kit	Cell culture medium
Immuno-isolation	Rapid High purity and specificity	Additional separation and purification are required Not suitable for mass generation	Cell culture medium Serum
UF	Rapid Simplicity of operation	Protein contamination Exosomes are damaged	Cell culture medium Serum Urine
Differential centrifugation	Rapid Mass production	Heterogeneity Easy to drain	Cell culture medium Serum Urine

Abbreviations: UC, ultracentrifugation; AF4, asymmetric flow field-flow fractionation; SEC, size-exclusion chromatography; UF, ultrafiltration.

**Table 3:** Exosome-based biochemical engineering strategy.

Strategy	Characters/Advantages	Applications for OA/Cartilage Repair
Modification of exosome-derived cells	Changing biological characteristics of exosomes: size, content, function, secretion, production efficiency, penetration	TGF-β1 [111] Overexpressing: miR-92a-3p [68], miR-320c [112] miR-135b [81] miR-95-5p [113]
3D culture		A more favorable environment for the proliferation of human synovial cells and the secretion of exosomes [114]
Deficiencies of Exosomes		GuiLu-ErXian Glue [115] Cartilage affinity peptide through lipid insertion [116] Buffer pH as a charge-reversal switch [117]
Exosomes Carrier	Good exosome retention and sustained release function as working platforms; Increasing the stability of content of exosomes;	Thiolated hyaluronic acid microgels [118] Photocrosslinking spherical gelatin methacryloyl hydrogel [119] Methacrylic anhydride-modified hyaluronic hydrogel [120] S-EXO-containing hydrogel microspheres [121] Injectable thermosensitive hydrogel [122]
3D printing	Designing more optimized 3D culture microenvironment; Designing scaffolds with more optimized geometric structure;	A photo-crosslinkable bioink containing different concentrations of silk methacrylate and polyethylene glycol diacrylate mixed with chondrocytes for biofabrication of 3D-bioprinted cartilage constructs [123] A modular hydrogel-based bioink containing microsphere-embedded chondrocytes for 3D printing multiscale scaffolds [124]

**Table 4:** Clinical trials registered in ClinicalTrials.gov regarding exosomes for osteoarthritis.

No	Title	ID	Conditions	Interventions
1	Mesenchymal Stem Cells Derived Exosomes in Osteoarthritis Patients	NCT06466850	Osteoarthritis, Knee	Biological: Exosome
2	Intra-articular Injection of MSC-derived Exosomes in Knee Osteoarthritis (ExoOA-1) (ExoOA-1)	NCT05060107	Osteoarthritis, Knee	Biological: Exosomes (sEVs)
3	Phase 1b Clinical Trial to Evaluate PEP and EUFLEXXA for Knee Osteoarthritis	NCT06463132	Osteoarthritis, Knee	Combination Product: PEP/Euflexxa Drug: Purified Exosome Product(PEP)
4	Intra-articular Injection of UC-MSC Exosome in Knee Osteoarthritis (EXO-OA01)	NCT06431152	Osteoarthritis, Knee	Biological: UC-MSC Exosomes (sEVs)

## Data Availability

No new data were created or analyzed in this study.
